# Syntaxin6 contributes to hepatocellular carcinoma tumorigenesis via enhancing STAT3 phosphorylation

**DOI:** 10.1186/s12935-024-03377-3

**Published:** 2024-06-04

**Authors:** Li Huang, Xiaoting Zhong, An Li, Fuping Tu, Miao He, Xueming Xu, Xiaohui Liu, Xiaoli Zeng, Jun Chi, Tian Tian, Chunli Wang, Xiangcai Wang, Jianming Ye

**Affiliations:** 1 Department of oncology, First Affiliated Hospital, Gannan Medical University, Ganzhou, China; 2 Jiangxi Clinical Medical Research Center for Cancer, Ganzhou, China; 3 Gannan Innovation and Translational Medicine Research Institute, Gannan Medical University, Ganzhou, China; 4 Department of critical medicine, First Affiliated Hospital, Gannan Medical University, Ganzhou, China; 5 128 Jinling Road, Ganzhou City, Jiangxi Province 341000 China

**Keywords:** STX6, HCC, JAK-STAT, STAT3, Tumorigenesis

## Abstract

**Background:**

Syntaxin6 (STX6) is a SNARE (Soluble N-ethylmaleimide-sensitive factor attachment protein receptors) protein complex located in the trans-Golgi network and endosomes, which is closely associated with a variety of intracellular membrane transport events. STX6 has been shown to be overexpressed in a variety of human malignant tumors such as esophageal, colorectal, and renal cell carcinomas, and participates in tumorigenesis and development.

**Methods:**

Based on clinical public database and clinical liver samples analysis, the expression of *STX6* in hepatocellular carcinoma (HCC) tissues was investigated. The effects of STX6 on proliferation, migration and invasion of HCC cell in vitro and in vivo were evaluated through gain- and loss-of-function studies. We further performed RNA-seq analysis and protein interactome analysis, to further decifer the detailed mechanisms of STX6 in the regulation of the JAK-STAT pathway in HCC.

**Results:**

*STX6* expression was upregulated in HCC tissues and its expression was highly correlated with the high histological grade of the tumor. STX6 promoted HCC cell proliferation, migration and invasion both in vitro and in vivo. Mechanistically, STX6 mediated tumor progression depending on promoting the activation of JAK-STAT signaling pathway. Receptor for activated protein kinase C (RACK1) as an essential adaptor protein mediating STX6 regulation of JAK-STAT pathway. Specifically, STX6 interacted with RACK1 and then recruited signal transducer and activator of transcription 3 (STAT3) to form a protein-binding complex and activates STAT3 transcriptional activity.

**Conclusions:**

This study provided a novel concept that STX6 exerted oncogenic effects by activating the STAT3 signaling pathway, and STX6 might be a promising therapeutic target for HCC.

**Supplementary Information:**

The online version contains supplementary material available at 10.1186/s12935-024-03377-3.

## Background

Primary liver cancer is the sixth most prevalent tumor worldwide and the fourth cause of cancer-related deaths [1]. In 2020, there were more than 900,000 new cases of liver cancer globally, of which the incidence rate in men was two to four times higher than that in women [2]. HCC is the main type of primary liver cancer, accounting for about 90% of all cases. HCC tends to occur in patients with underlying liver diseases, most commonly due to hepatitis B or C virus (HBV or HCV) infection or alcohol abuse [3]. Most patients with HCC exhibit poor early clinical manifestations and are diagnosed at an intermediate to advanced stage, resulting in a poor prognosis and a mortality rate close to the global incidence rate, with a current five-year survival rate of only 18% [4]. The treatment of HCC includes surgical treatment, transcatheter arterial chemoembolization, radiofrequency ablation, radioimmunoassay, and targeted drug therapy. In recent years, despite the continuous progress of treatment strategies, the global mortality rate of HCC has continued to rise [5]. Therefore, it is necessary to further study the detailed mechanism of HCC and to explore new therapeutic targets.

Soluble N-ethylmaleimide-sensitive factor attachment protein receptors (SNAREs) are the key mediators of membrane fusion. The conserved central coiled-coil SNARE motif with 60–70 residues mediates the interaction between vesicle SNAREs (v-SNAREs) and target SNAREs (t-SNAREs) [6]. STX6 is a t-SNARE encoded by the *STX6* gene located on human chromosome 1. STX6 contains 255 amino acids, including a C-terminal hydrophobic anchor and two coiled-coil domains, of which the C-terminal contains a SNARE motif [7]. STX6 is distributed in the trans-Golgi network and endosomes, involved in the formation of a variety of complexes, and is related to intracellular and extracellular transport functions, such as endocytosis, recycling, forward and reverse transport [8, 9]. For example, STX6 plays an important role in immune cell exocytosis by promoting the secretion and transport of TNF-α in activated macrophages [8] and mediating the release of neutrophil inflammatory granules [10]. STX6 also affects angiogenesis by maintaining the endocytosis cycle of α5β1 integrin [11]. Other studies have shown that STX6 is involved in a variety of neurological diseases by regulating nerve growth factor-dependent neurite growth, such as Parkinson’s disease, Alzheimer ‘s disease and prion disease [7, 12, 13]. In addition, STX6 has been found to be associated with the progression and prognosis of a diverse range of tumors. In esophageal cancer, STX6 expression is upregulated, and its expression level is closely related to tumor size, histological differentiation, lymph node metastasis and depth [14]. In renal cell carcinoma, high expression of STX6 is associated with decreased survival of patients [15]. A recent study have found that in colorectal cancer, knocking down STX6 blocks the cell cycle and inhibits cell proliferation, migration and invasion [16].However, the specific function and mechanism of STX6 in hepatocellular carcinoma need to be further clarified. Some reports have documented the abnormal overexpression of *STX6* in some human tumors such as HCC, colorectal cancer, pancreatic ductal adenocarcinoma, as well as esophageal cancer [14, 16–18], and have found that STX6 is involved in the development of these tumors. Overexpression of *STX6* in HCC tissues was revealed to be significantly associated with poor prognosis of patients [19]. However, the regulatory mechanism by which STX6 plays an oncogenic role in HCC requires further exploration.

In this study, we found that *STX6* was up-regulated in HCC tissues compared with paracancerous tissues, and it promoted cell proliferation, migration and invasion of HCC cells both in vitro and in vivo by regulating the phosphorylation of signal transducer and activator of transcription 3 (STAT3). Our results suggest that STX6 is a key regulator in the pathogenesis of HCC and may serve as a potential therapeutic target for HCC.

## Methods

### Human liver samples

Patients with postoperative pathologic diagnosis of HCC in the First Affiliated Hospital of Gannan Medical University were included in this study. None of the enrolled patients received preoperative local treatment (including liver transplantation, ablation, cryotherapy, radiotherapy, etc.) and systematic treatment. Patients with active autoimmune disease requiring systemic therapy (including immunomodulatory drugs, corticosteroid drugs, or immunosuppressive drugs), severe cardiovascular or cerebrovascular disease, previous allogeneic stem cell or solid organ transplantation, or malignant tumors other than primary hepatocellular carcinoma (besides cured confined tumors) within 5 years were excluded. Surgically resected fresh HCC tissue and matching adjacent non-tumor tissue samples were rapidly frozen with liquid nitrogen for molecular biological detection. Clinical information was available. Ethical approval for this study was obtained from the Ethical Review Committee of the First Affiliated Hospital of Gannan Medical University, and informed consent was obtained from all patients before treatment. The study was conducted in accordance with the Declaration of Helsinki.

### Animals and tumor xenograft model

All animal experimental protocols were approved by the Animal Care and Use Committee of the First Affiliated Hospital of Gannan Medical University. Experiments were performed in accordance with the Guide for the Care and Use of Laboratory Animals published by the National Institutes of Health. BALB/c nude mice were housed in laminar-flow cabinets under specific pathogen-free conditions.BALB/c nude mice were kept for 5–7 days as an adaptation period before being used in experiments. 5 × 10^6^ HepG2 or Huh7 cells infected with sh*STX6* or shRNA resuspended in 200µL PBS and matrix gel were injected subcutaneously into the right axilla of 6-week-old BALB/c nude mice (*n* = 6 mice per group). The body weight of each nude mice was monitored and tumor size (length and width) were measured utilizing Vernier caliper every 2 or 3 days [20]. Tumor volume was determined using the following formula: volume = 0.5 × length × width^2^. Mice were sacrificed at 30 or 34 days post-injection. At the end of the experiment, the tumors were collected, weighed, and photographed.

### Gene expression and survival analysis

Transcriptome sequencing data and clinical information of human HCC samples and non-tumor samples were downloaded from TCGA (The Cancer Genome Atlas) databases, and differential gene expression analysis between cancer and non-tumor samples was carried out by DESeq2, an *R* package standardized based on negative binomial distribution. The standardized data were then subjected to grade classification, pathological stage correlation analysis, and survival analysis. The *t*-test was used to analyze the correlation between *STX6* expression and grading and staging in tumor clinical information. Data were visualized using *R* package ggplot2. For survival curves and data visualization, the HCC clinical samples were divided into two groups according to the *STX6* expression, and the results were obtained using *R* package survminer.

### RNA extraction and real-time PCR

Total RNA was extracted from HCC tissues and cells using TRIzol reagent and following the manufacturers’ guidelines. The RNA samples were then reverse transcribed into complementary DNA (cDNA) using oligo dT primers and reverse transcriptase. Real-time qPCR was performed using SYBR Green PCR Master Mix following the procedure provided by the manufacturer. *β-actin* was used as the internal reference. STX6-Forward: AGGAACAGGCAGTTATGTTGGA. STX6-Reverse: TATGCAGGAGGAACTCGCAC. β-actin-Forward: CATGTACGTTGCTATC-CAGGC. β-actin-Reverse: CTCCTTAATGTCACGCACGAT.

### Western blotting analysis

HCC tissues or cells were fully lysed in RIPA buffer and then centrifuged at 13,300 *g* for 15–30 min. The concentration of the samples was determined using BCA Protein Assay Kit (23,225, Thermo Fisher Scientific, USA). The protein samples were then subjected to polyacrylamide gel electrophoresis and transferred to a polyvinylidene difluoride (PVDF) membrane. The membranes were blocked with 5% skimmed milk at room temperature for 1 h. After washing, the corresponding primary antibodies were incubated with PVDF at 4℃ overnight. Subsequently, PVDF membranes were incubated with the corresponding secondary antibodies (115-035-003 or 111-035-003, Jackson ImmunoResearch Laboratories, USA) for 1 h at room temperature. Western blotting Substrate kit (BLWB021-100ML, BioLight, China) and the ChemiDoc™ XRS + Imaging System (Bio-Rad, USA) were used to detect and visualize the proteins. The used primary antibodies were as follows: GAPDH (Proteintech, 60004-1), STX6 (Abclonal, A19813), ACTIN (Abclonal, AC026), PCNA (Biolight, CP00140HuA10), CYCLIND1 (Abclonal, A22104), E-CAD (Biolight, RMAP0043M1), N-CAD (Abclonal, A3045), p-STAT3 (CST, 9145 S), STAT3(CST, 12,640 S), RACK1 (CST, 5432 S).

### Immunohistochemistry

Tumor tissue specimens of HepG2 sh*STX6*- and HepG2 shRNA-derived xenograft tumor were fixed in 4% paraformaldehyde, dehydrated in ethanol and paraffin-embedded. Sections were cut using a sectioning machine and then deparaffinized in xylene and rehydrated in graded alcohol. Tissue sections were stained by IHC. 5% goat serum was used to seal the tissue sections, and then the sections were incubated with PCNA (Abclonal, A12427), Ki67 (Abclonal, A23722) or STX6 (Abclonal, A19813) antibody overnight at 4℃, followed by incubation with biotin-labeled secondary antibody. Sections were visualized by 3,3’-diaminobenzidine (DAB) substrate and then photographed with a microscope.

### Plasmid construction

To obtain the plasmid overexpressing *STX6*, the primers were designed and synthesized online using Primer5 software, and then the STX6 cDNA was amplified by PCR using human cDNA as a template. The amplified fragment was cloned into the vector pHAGE-Flag using recombinase. Finally, the plasmid was extracted and then enzymatically identified. To construct the *STX6* knockdown plasmid, upstream primers and downstream primers targeting the RNA interference sequence of STX6 gene were designed and synthesized, and the synthesized upstream primers and downstream primers were annealed. The annealed oligonucleotide strand was ligated to the digested linear vector using a double-enzymatic vector, and finally the target gene expression detection was performed.

### Lentivirus construction and transfection

For lentiviral infection, HEK-293T cells were first vaccinated in 15-cm dishes, and transfection was performed the next day when the cells were 80–90% spread. Recombinant plasmid (pMD2.G), lentiviral helper plasmid (psPAX2), and lentiviral target plasmid were co-transfected into the cells in the presence of polyethyleneimine transfection reagent. The medium was changed 6 h after transfection, and the viral supernatant was harvested 48 h later. Then, the obtained lentiviruses were added to the target cells in the presence of polyglutamine (8 µg/mL), respectively. 48 h later, the infected cells were screened with puromycin (2 µg/mL).

### Cell lines and culture

Human 293T cell, human hepatocellular carcinoma cell lines Huh7 and HepG2 were purchased from ATCC. These cells were cultured in high-glucose DMEM medium containing 10% fetal bovine serum, 100 units/mL penicillin and 100 g/mL streptomycin in a 37℃, 5% CO_2_ incubator. During incubation, the cell medium were changed three times a week to guarantee optical growth, and the cells were passaged at the ratio of 1:3, when the confluence reached 80–90%. To evaluate if the role of STX6 in HCC depends on STAT3 activity, we applied an inhibitor of STAT3 (Stattic, 10 µM) in STX6 overexpressed cells and performed the rescue assays in vitro.

### Cell counting kit-8 assay

Cell Counting Kit-8 (CCK-8) assay was performed according to the instructions of CCK-8 kit. Huh7 cells and HepG2 cells were inoculated in 96-well plates at a density of 3,000 cells per well. Cell proliferation was measured at 0, 24, 48, 72, 96 and 120 h after cell attachment. When evaluated, an equal volume of CCK-8 solution to 10% of total cell medium was added to each well, and the plate was incubated at 37 °C for an additional 2 h under controlled conditions. Optical density at the wavelength of 450 nm was measured to assess cell viability.

### Colony formation assay

2,000 cells per well were inoculated into six-well plates for colony formation assays. After 9 days of culture, the cells were gently washed twice in PBS, which were subsequently fixed using 4% paraformaldehyde for 15 min at room temperature and stained with 0.1% crystal violet for 15 min at 37℃. Visible colonies were photographed and counted by ImageJ software.

### Transwell assay

For migration experiments, HCC cells were seeded into the upper chamber of the transwell plate (3422, Corning, New York, USA), the upper chamber was filled with a cell suspension with serum-free DMEM medium and the lower chamber was filled with medium containing 20% fetal bovine serum. HCC cells were inoculated into the upper chamber of Transwell plates. After incubation for the appropriate time, the cells were fixed with 4% paraformaldehyde and stained with 0.1% crystal violet. For invasion assays, cells were inoculated into the upper chamber after coated with Matrigel.

### Wound healing assay

HCC cells were inoculated in 24-well plates (1 × 10^6^/well), and after the cells grew to 90% fusion, a linear scratch was made on the cell monolayer with a 200 µL sterile pipette tip. The scratched wounds were then slowly rinsed 3 times with phosphate buffer solution (PBS), and then medium containing 2.5% FBS was added, and the scratched wounds were observed with a phase contrast microscope. Cell images were acquired under the microscope before and 48 h after stimulation, respectively. The area of each scratch wound was determined using Image J software.

### RNA-sequencing and correlative analysis

Total RNA was extracted as the previous description and cDNA libraries were constructed. Sequencing was conducted using a BGISEQ 500 instrument and building a single-end sequencing of libraries.

### Gene set enrichment analysis

Gene set enrichment analysis (GSEA) was performed using GSEA software (http://software.broadinstitute.org/gsea/index.jsp). The gene were sorted according to the level of differential expression, and gene sets based on the gene ontology database were examined to determine whether they were concentrated at the top or bottom of the sorting list to investigate the overall expression changes. Java GSEA was used to perform GSEA with the “Signal2Noise” metric. Gene sets with P values less than 0.05 and FDR less than 0.25 were considered statistically significant.

### Kyoto Encyclopedia of genes and genomes enrichment analysis

All differentially expressed genes (DEGs) were analyzed by Fisher’s exact test for Kyoto Encyclopedia of Genes and Genomes (KEGG) pathway enrichment and download KEGG pathway annotations for all genes in the reference genome from the KEGG database.

### Statistical analysis

All data are expressed as mean ± standard deviation (mean ± SD) and were analyzed using Statistical Product and Service Solutions (SPSS software; version 19.0; SPSS, Inc., Chicago, IL) software for statistical analysis. For normally distributed data, two-tailed Student’s *t*-tests were used for comparisons between two groups and one-way ANOVA was used for comparisons between multiple groups, followed by Bonferroni analysis (for data that satisfy chi-square) or Tamhane’s T2 analysis (for data showing heteroscedasticity). For data sets with skewed distributions, nonparametric statistical analyses were performed using the Mann-Whitney U test and the Kruskal-Wallis test, with differences considered statistically significance at *P* < 0.05.

## Results

### STX6 exhibits oncogenic role in HCC

**Fig. 1:**
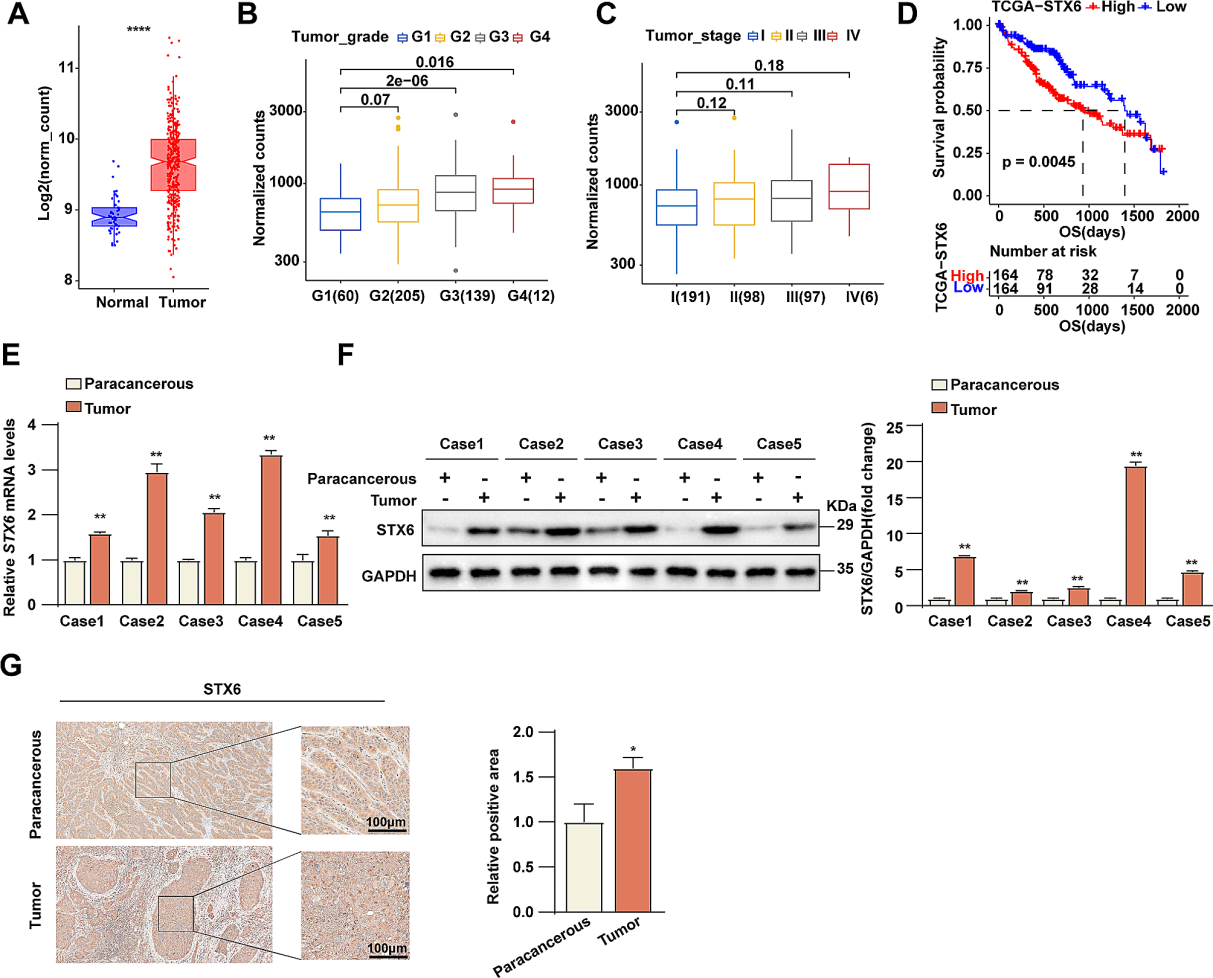
STX6 is upregulated in HCC patients and correlates to HCC progression. (**A**) Results of *STX6* expression level analysis in human HCC tissues (*n* = 347) and normal tissues (*n* = 50) in TCGA databases. (**B**) *STX6* expression levels in HCC samples of different histologic grades in TCGA databases. Histologic grading grade I, *n* = 60; grade II, *n* = 205; grade III, *n* = 139; grade IV, *n* = 12. (**C**) *STX6* mRNA levels in HCC samples with different TNM stages in TCGA databases. Stage I, *n* = 191; Stage II, *n* = 98; Stage III, *n* = 97; Stage IV, *n* = 6. (**D**) Kaplan-Meier overall survival curves of HCC patients with high and low *STX6* expression in the TCGA database. (**E**) *STX6* mRNA levels in paired HCC and paracancerous tissues. *β-actin* was used to normalize. Data was given as mean ± SD. (**F**) The WB and quantification results of STX6 in HCC tissues and paracancerous tissues. GAPDH served as the loading control. Data was given as mean ± SD. (**G**) Results of IHC analysis of STX6 in HCC tissues and paracancerous tissues. Data was given as mean ± SD. For statistical analysis, two-tailed Student’s *t*-test was used in **E**-**G**. * *P* < 0.05; ** *P* < 0.01

To investigate the potential role of STX6 in HCC, we first analyzed the expression levels of *STX6* in human HCC tissues and normal tissues using TCGA (The Cancer Genome Atlas) databases. The results showed that the expression of *STX6* was significantly upregulated in tumor tissues compared with normal tissues **(**Fig. 1A**)**. To further study the role of *STX6* in HCC progression, we examined the correlation between *STX6* expression levels and clinicopathologic features of HCC. The data showed that the high *STX6* expression was closely correlated to advanced histological grading of the tumor **(**Fig. 1B, C**)**. Secondly, the overall survival (OS) between HCC patients with high and low expression of *STX6* was analyzed to explore the relationship between *STX6* and prognosis of HCC patients. It’s found that high expression of *STX6* did not result in reduction of the patients’ survival rate **(**Fig. 1D**)**. Next, to further confirm the correlation between *STX6* and HCC, we collected human HCC tissues and paired paracancerous tissues to detect the expression of STX6 by RT-PCR and WB techniques. The results showed that both mRNA and protein levels of STX6 were significantly up-regulated in HCC tissues compared with paracancerous tissues **(**Fig. 1E, F**)**. These data suggest that STX6 is associated with the progression of HCC and may play an important role in the development of HCC. In addition, the IHC analysis of clinical HCC samples showed that the expression of STX6 was significantly higher in tumors than in paracancerous tissues, which was consistent with the analysis results of the TCGA database **(**Fig. 1G**)**. These data suggest that STX6 was overexpressed in HCC and might serve as an oncogene in HCC progression.

### STX6 promotes HCC cell proliferation in vitro

**Fig. 2:**
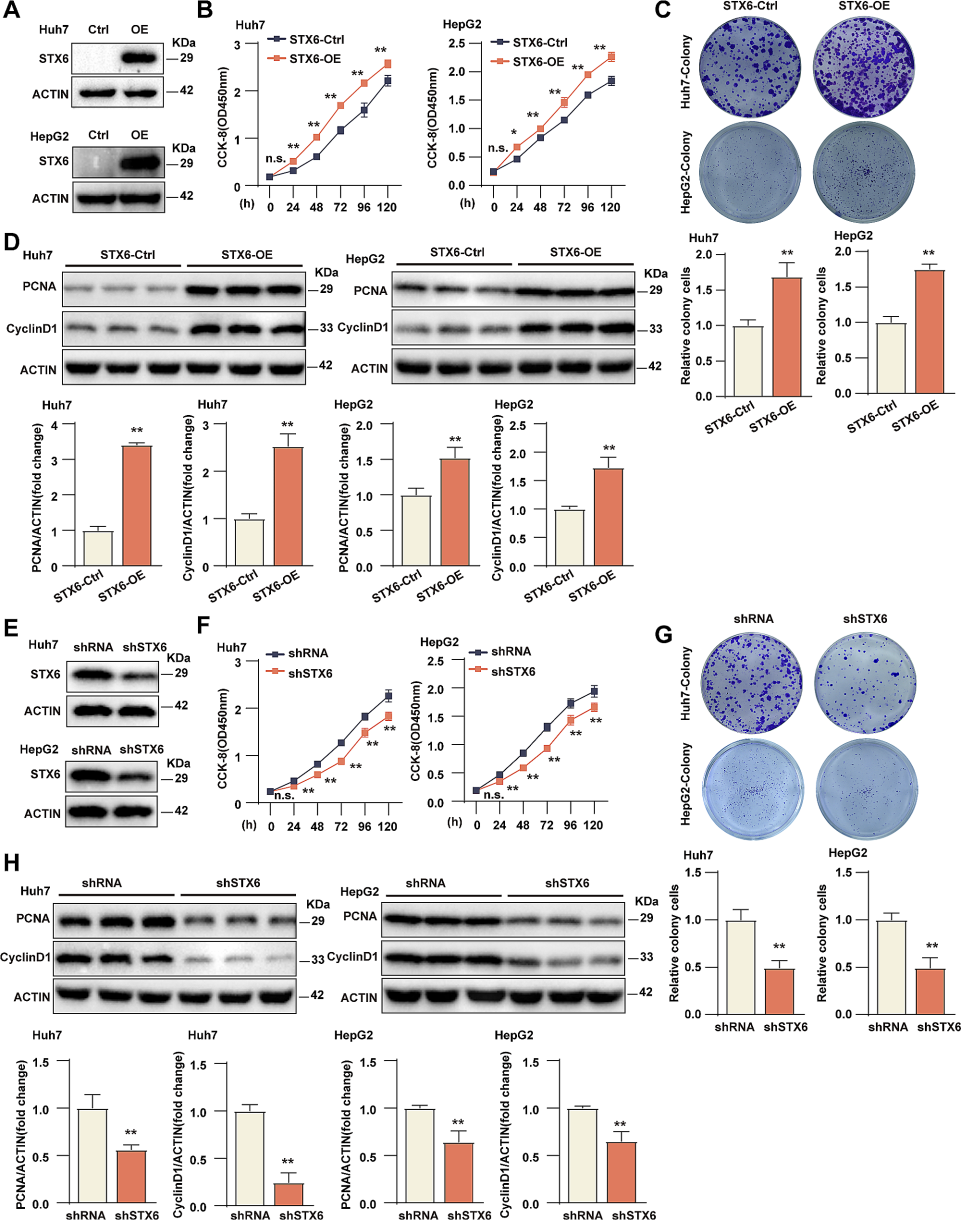
STX6 increases the proliferative capacity of the HCC cells. (**A**) STX6 protein expression level in Huh7/HepG2 STX6-OE and corresponding control cells. (**B**) Results of CCK8 experiments in Huh7/HepG2 STX6-OE and corresponding control cell groups. *n* = 5 per group. (**C**) The representative images and quantification results of Huh7/HepG2 STX6-OE and corresponding control cells in cell colony formation assay. *n* = 3 per group. (**D**) Protein levels and quantification results of proliferation-related molecules PCNA and cyclin D1 in Huh7/HepG2 STX6-OE and corresponding control cells. *n* = 3 per group. β-actin served as the loading control. (**E**) STX6 protein expression level in Huh7/HepG2 sh*STX6* and corresponding control cells. (**F**) Results of CCK8 experiments in Huh7/HepG2 sh*STX6* and corresponding control cell groups. *n* = 5 per group. (**G**) The representative images and quantification results of Huh7/HepG2 sh*STX6* and corresponding control cells in cell colony formation assay. *n* = 3 per group. (**H**) Protein levels and quantification results of proliferation-related molecules PCNA and cyclin D1 in Huh7/HepG2 sh*STX6* and corresponding control cells. *n* = 3 per group. β-actin served as the loading control. Data was given as mean ± SD. For statistical analysis, the two-tailed Student’s *t*-test was used in **B**-**D** and **F**-**H**. n.s. indicates no significant difference; * *P* < 0.05; ** *P* < 0.01

To explore the biological function of STX6 in HCC, we constructed Huh7/HepG2 cell line overexpressing STX6 (STX6-OE) by lentiviral system and verified its efficacy by WB analysis **(**Fig. 2A**)**. The CCK-8 assay and colony formation assay were performed to examine the effects of STX6 on Huh7/HepG2 cell viability and proliferation efficiency. The CCK-8 assay showed that overexpression of STX6 promoted Huh7/HepG2 cell viability **(**Fig. 2B**)**. The colony formation assay showed that in Huh7/HepG2 cells, the number of clones in the STX6-OE group was significantly more than that in the control group **(**Fig. 2C**)**. Furthermore, the expression of proliferation-associated molecules PCNA (Proliferating cell nuclear antigen) and cyclin D1 (Cell cycle protein D1) were remarkably up-regulated in the STX6-OE group compared with the control **(**Fig. 2D**)**. On the other hand, the STX6 knockdown Huh7/HepG2 cell lines (sh*STX6*) by shRNA-mediated gene silencing were also constructed, and the effectiveness of STX6 knockdown was also confirmed **(**Fig. 2E**)**.The CCK-8 and the colony formation assay showed that comparing with the control group, the Huh7/HepG2 cells in the sh*STX6* group significantly reduced cell viability and proliferation ability **(**Fig. 2F, G**)**, and the expression levels of proliferation-related molecules PCNA and cyclin D1 were decreased obviously **(**Fig. 2H**)**. All these indicated that STX6 promoted the proliferation of HCC cells.

### STX6 promotes HCC cell migration and invasion in vitro

**Fig. 3:**
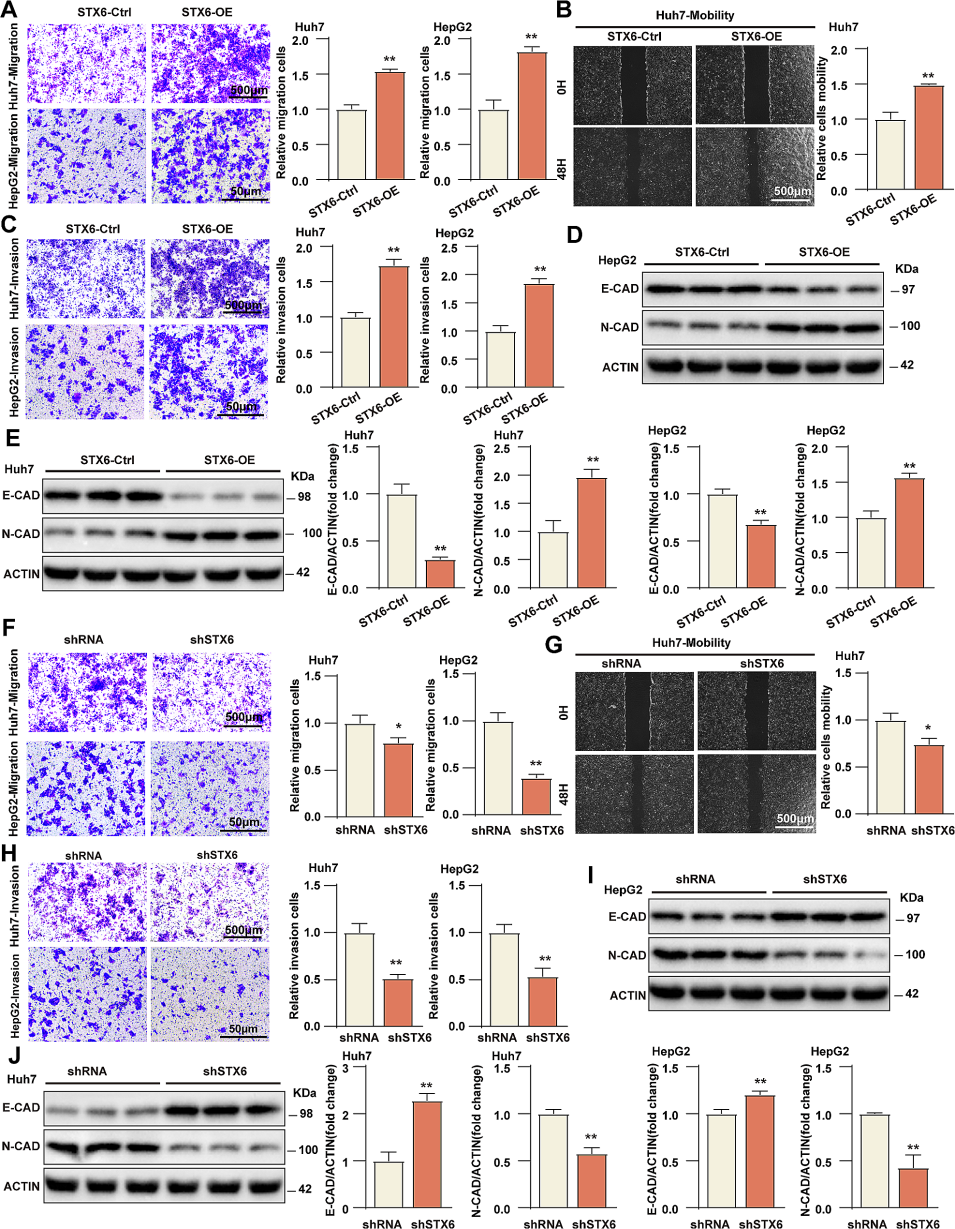
STX6 promotes migration and invasion of HCC cells. (**A**) The representative images and quantification results of Transwell migration assay of Huh7/HepG2 STX6-OE and corresponding control cells. *n* = 3 per group. (**B**) The representative images and quantification results of Huh7 STX6-OE and corresponding control cells. *n* = 3 per group in wound healing assay. (**C**) The representative images and quantification results of Huh7/HepG2-STX6-OE and corresponding control cells in Transwell invasion assay. *n* = 3 per group. (**D**-**E**) Protein levels and quantification results of metastasis-related E-cadherin and N-cadherin in Huh7/HepG2 STX6-OE and corresponding control cells. *n* = 3 per group. (**F**) The representative images and quantification results of Huh7/HepG2 sh*STX6* and corresponding control cells in Transwell migration assay. *n* = 3 per group. (**G**) The representative images and quantification results of wound healing assay of Huh7 sh*STX6* and corresponding control cells. *n* = 3 per group. (**H**) The representative images and quantification results of Transwell invasion assay of Huh7/HepG2 sh*STX6* and corresponding control cells. *n* = 3 per group. (**I**-**J**) Protein levels and quantification results of metastasis-related E-cadherin and N-cadherin in Huh7/HepG2 sh*STX6* and corresponding control cells. *n* = 3 per group. β-actin served as the loading control. Data was given as mean ± SD. For statistical analysis, the two-tailed Student’s *t*-test was used in **A**-**C**, **E**-**H**, and **J**. * *P* < 0.05; ** *P* < 0.01

To further evaluate the effects of STX6 on HCC cell migration and invasion, we performed Transwell migration assay, wound healing assay and Transwell invasion assay. The results showed that overexpression of STX6 dramatically promoted the migration of Huh7 and HepG2 cells **(**Fig. 3A**)**. In the wound healing assay, the migration ability of Huh7 cells overexpressing STX6 was clearly enhanced at 48 h comparing with the control cells **(**Fig. 3B**)**. The Transwell invasion assay revealed that the invasion ability of Huh7 and HepG2 cells overexpressing STX6 was increased considerably compared with the control group **(**Fig. 3C**)**. In Huh7 and HepG2 cell lines, the expression of metastasis-promotive factor N-cadherin was enhanced in the STX6-OE group, while the expression of metastasis-inhibitory E-cadherin was diminished relative to the control group **(**Fig. 3D, E**)**. On the contrary, Transwell migration assay demonstrated that STX6 knockdown decreased Huh7/HepG2 cell migration **(**Fig. 3F**)**, and the wound healing assay showed that STX6 knockdown inhibited Huh7 cell migration **(**Fig. 3G**)**. The results of Transwell invasion assay exhibited that the invasion ability of Huh7/HepG2 cells transfected with sh*STX6* was severely weakened **(**Fig. 3H**)**, which was also confirmed by the expression assay of metastasis-related molecules **(**Fig. 3I, J**)**. Taken together, it’s concluded that STX6 promoted HCC cell migration and invasion in vitro.

### STX6 promotes tumorigenic behavior of HCC in vivo

**Fig. 4:**
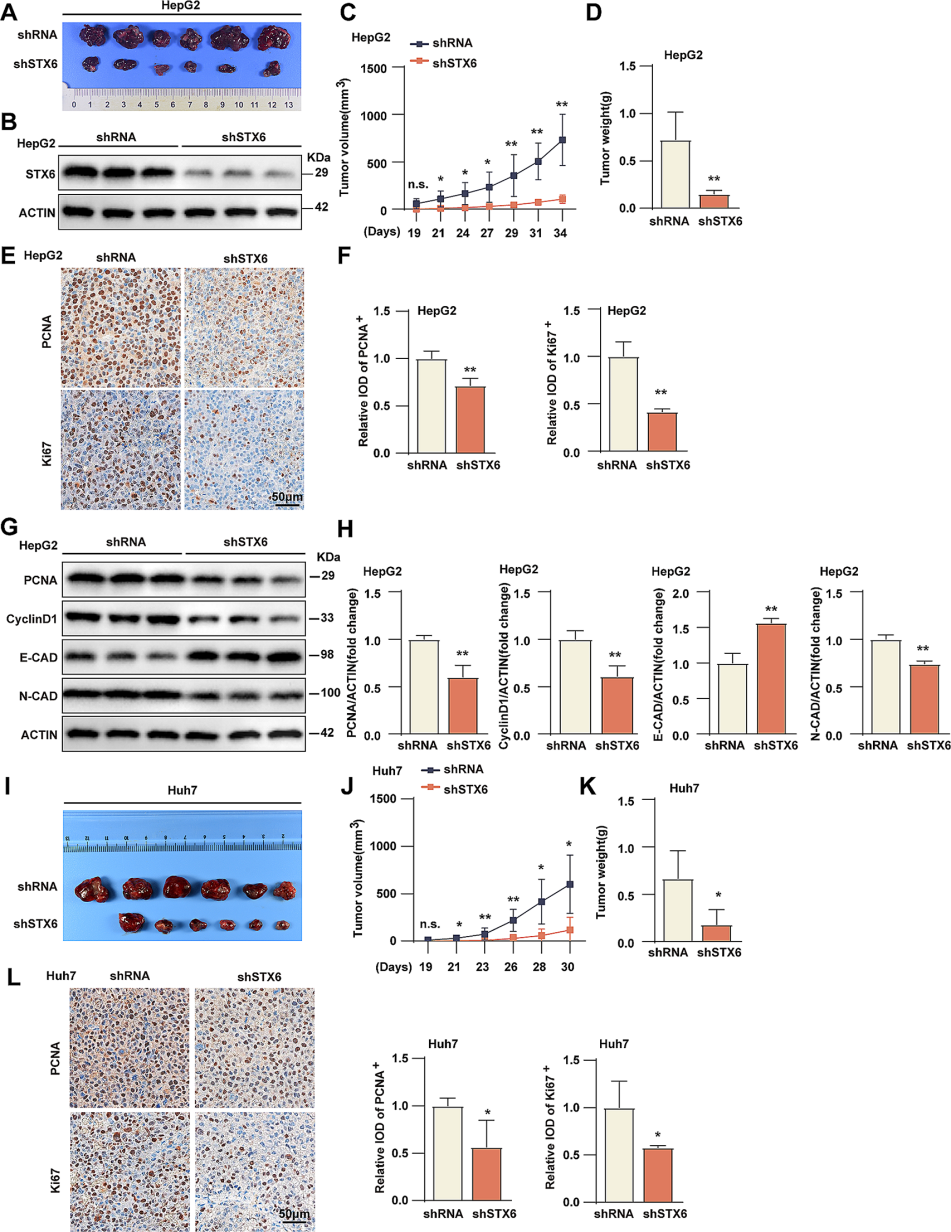
STX6 promotes HCC tumorigenic behavior in vivo. (**A**) Representative images of HepG2 shRNA- and HepG2 sh*STX6*-derived xenograft tumors. *n* = 6 per group. (**B**) STX6 expression in HepG2 shRNA- and HepG2 sh*STX6*-derived xenograft tumors. (**C**) Results of tumor volume analysis at different days after HepG2 cells’ injection. *n* = 7 per group. (**D**) Tumor weight of HepG2 shRNA- and HepG2 sh*STX6*-derived xenograft tumors. *n* = 7 per group. (**E**-**F**) The representative images (**E**) and quantification results (**F**) of immunohistochemical staining of PCNA and Ki-67 in the HepG2 shRNA- and HepG2 sh*STX6*-derived xenograft tumors. *n* = 4 per group. (**G**-**H**) Protein levels (**G**) and quantification results (**H**) of PCNA, cyclin D1, E-cadherin and N-cadherin in HepG2 shRNA- and HepG2 sh*STX6*-derived xenograft tumors. *n* = 3 per group. β-actin served as the loading control. Data was given as mean ± SD. (**I**) Representative images of Huh7 shRNA- and Huh7 sh*STX6*-derived xenograft tumors. *n* = 6 per group. (**J**) Results of tumor volume analysis at different days after Huh7 cells’ injection. *n* = 7 per group. (**K**) Tumor weight of Huh7 shRNA- and Huh7 sh*STX6*-derived xenograft tumors. *n* = 7 per group. (**L**) The representative images and quantification results of immunohistochemical staining of PCNA and Ki-67 in the Huh7 shRNA- and Huh7 sh*STX6*-derived xenograft tumors. *n* = 4 per group. For statistical analysis, the two-tailed Student’s *t*-test was used in **C**, **D**, **F**, **H** and **J**-**L**. n.s. indicates no significant difference; * *P* < 0.05; ** *P* < 0.01

To investigate the role of STX6 in tumorigenic behavior in vivo, we injected STX6 knockdown HepG2 cells and its corresponding control cells subcutaneously into BALB/c nude mice. We found that the tumor size in the shSTX6 group was smaller than the control group (Fig. 4A). The low expression of STX6 in the shSTX6 group was verified by WB experiments (Fig. 4B). Tumor growth was observed from 19 days after injection, and the tumor volume of the sh*STX6* group increased much slower than control group **(**Fig. 4C**)**. The tumor weight was also lower than the control group **(**Fig. 4D**)**. The immunohistochemistry staining of PCNA and Ki-67 protein in different groups of tumors indicated that STX6 knockdown decreased PCNA and Ki-67 expression in vivo **(**Fig. 4E, F**)**. Consistently, WB analysis of tumor tissues showed that STX6 knockdown suppressed the expression of PCNA, cyclin D1 and N-cadherin, but promoted the expression of E-cadherin **(**Fig. 4G, H**)**. The similar conclusion was obtained in STX6 knockdown Huh7 cells **(**Fig. 4I, J, K, L**)**. Taking all into account, it’s proved that STX6 promoted tumorigenic behavior of HCC in vivo.

### STX6 promotes the phosphorylation of STAT3

**Fig. 5:**
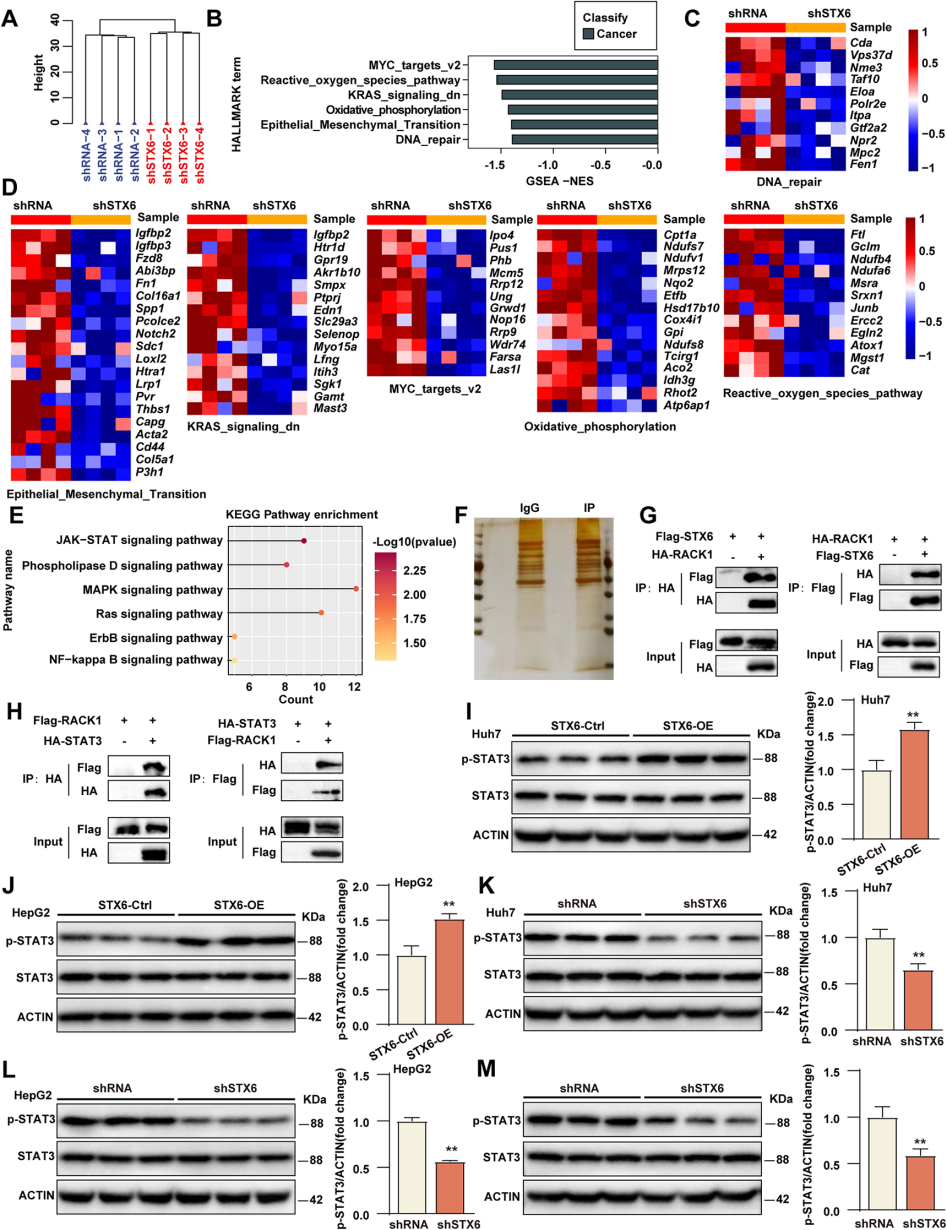
STX6 regulates the STAT3 signaling pathway. (**A**) Cluster analysis of RNA-Seq results from HepG2 STX6 knockdown and control cell lines. (**B**) Gene set enrichment analysis of RNA-Seq results from HepG2 STX6 knockdown and control cell lines. (**C**-**D**) Gene expression levels associated with MYC, reactive oxygen species pathway, KRAS signaling pathway, oxidative phosphorylation, EMT, and DNA repair in HepG2 STX6 knockdown and control cell lines. (**E**) KEGG pathway enrichment analysis in HepG2-sh*STX6* cells. (**F**) IP-MS experiment was used to identify the potential targets of STX6 in Falg-STX6-overexpressed HepG2 cells. (**G**) Co-IP analysis of the interaction between STX6 and RACK1 from IP-MS in 293T cells. (**H**) Co-IP analysis of the interaction between STX6 and STAT3 in 293T cells. (**I**-**J**) Total and phosphorylated STAT3 protein levels in Huh7/HepG2 STX6-OE and its control cells. β-actin served as the loading control. (**K**-**L**) Total and phosphorylated STAT3 protein levels in Huh7/HepG2 sh*STX6* and its control cells. β-actin served as the loading control. (**M**) Total and phosphorylated STAT3 protein levels in HepG2 shRNA- and HepG2 sh*STX6*-derived xenograft tumors. β-actin served as the loading control. Data was given as mean ± SD. For statistical analysis, the two-tailed Student’s *t*-test was used in **I**-**M**. * *P* < 0.05; ** *P* < 0.01

To explore the underlying mechanism by which STX6 exerts its function, we performed RNA sequencing (RNA-Seq) analysis using STX6 knockdown HepG2 cells and its control cells. Principal component analysis and hierarchical clustering clearly separated the samples into two clusters incorporating the sh*STX6* cells and control cells, respectively **(**Fig. 5A**)**. To further explore the changes in signaling pathways between the two clusters, we performed gene set enrichment analysis (GSEA) and found that STX6 knockdown inhibited MYC, reactive oxygen species pathway, KRAS signaling pathway, oxidative phosphorylation, EMT (Epithelial-mesenchymal transition), and DNA repair pathway **(**Fig. 5B, C, D**)**, all of which were associated with tumorigenic characteristics, and implied STX6 exerted promotive effects on HCC progression on the whole. To further explore the downstream mechanisms, an unbiased KEGG analysis showed that the JAK-STAT signaling pathway was the most significantly altered pathway regulated by STX6 knockdown **(**Fig. 5E**)**. To systematically identify the direct and key regulators of STX6, we constructed Flag-tagged STX6-overexpressing HepG2 cells and conducted IP coupled with mass spectrometry (IP-MS) using an anti-Flag antibody and control IgG **(**Fig. 5F**)**. We firstly screened proteins by sorting their peptide spectrum scores, and then searched for potential candidates related to JAK-STAT signaling pathway. Among these candidates, we identified that the adapter protein receptor for activated protein kinase C (RACK1), which plays a central role in early signaling through the JAK-STAT pathway, might be a linker between STX6 and JAK-STAT pathway [21]. RACK1 can interact with the classical elements of JAK-STAT pathway, such as signal transducer and activator of transcription 3 (STAT3) [22]. Abundant reports indicate that STAT3 is among the STAT transcription factor family and a critical factor regulating HCC tumor progression [23–25], thus we hypothesized that STX6 may alter the JAK-STAT signaling pathway by regulating STAT3. Further IP experiments were carried out to validate the interaction of these candidate proteins with STX6 and those data confirmed the interaction of STX6 and RACK1 or RACK1 and STAT3 **(**Fig. 5G, H**)**. As a classical signal responder, STAT3 serves as a transcriptional factor once activating as a phosphorylation status. Thus, we performed WB analysis and data showed that phosphorylated STAT3 (p-STAT3) not the total STAT3 levels were increased in Huh7/HepG2 cells overexpressing STX6 **(**Fig. 5I, J**)**, but decreased in Huh7/HepG2 cells knocking down STX6 **(**Fig. 5K, L**)**in vitro. Consistently, the expression of p-STAT3 in tumor tissues derived from sh*STX6* HepG2 xenografts was also decreased **(**Fig. 5M**)**. All these concluded that STX6 might function through promoting STAT3 phosphorylation.

### Phosphorylated STAT3 mediated the promotive role of STX6 in HCC progression

**Fig. 6:**
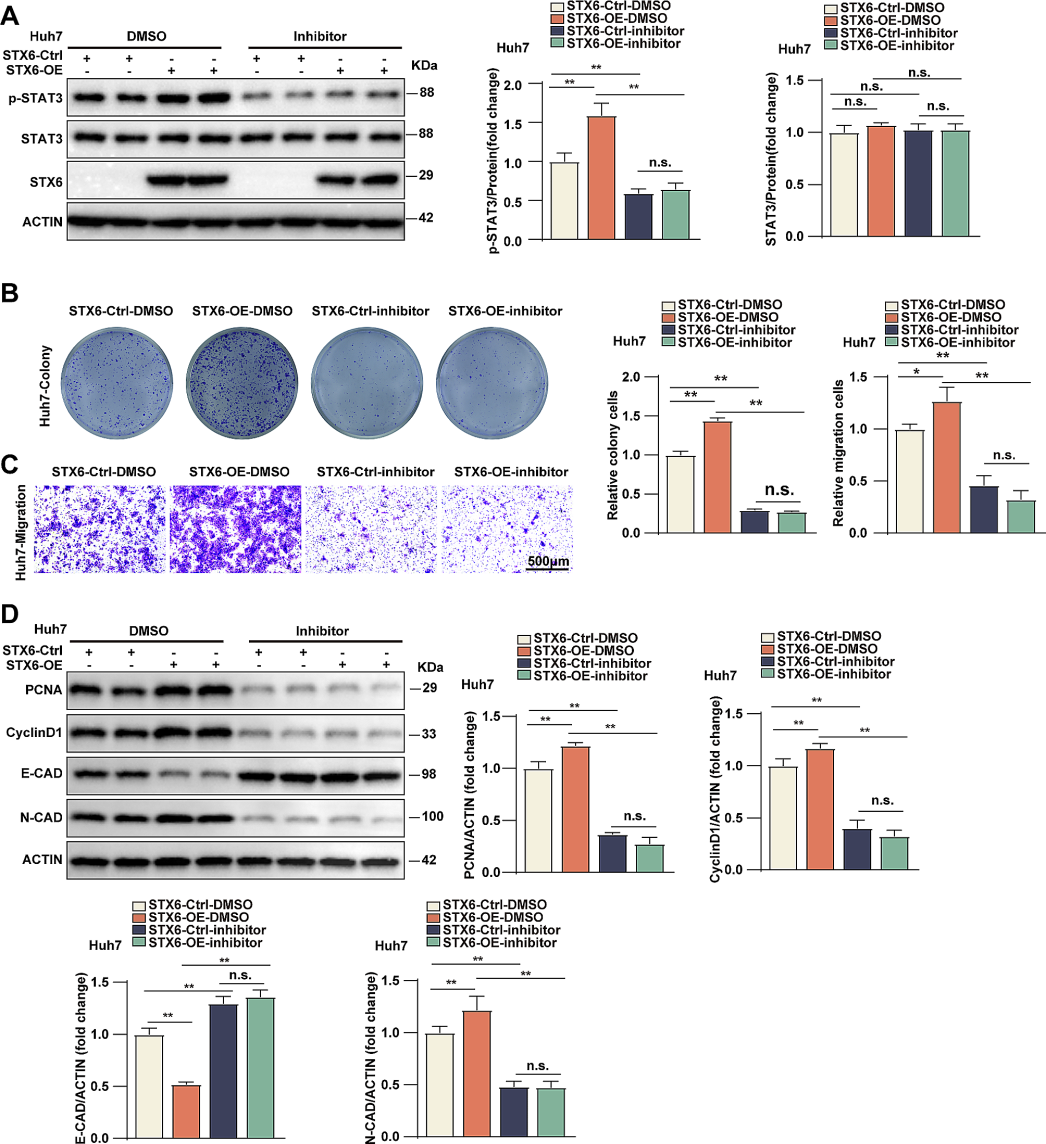
STAT3 phosphorylation mediated the promotive role of STX6 in HCC progression. (**A**) Protein levels and quantification results of total and phosphorylated STAT3 in control and Huh7 STX6-OE cells treated with p-STAT3 inhibitor or DMSO. β-actin served as the loading control. (**B**) The representative images and quantification results of control and Huh7 STX6-OE cells treated with p-STAT3 inhibitor or DMSO in colony formation assays. *n* = 3 per group. (**C**) The representative images and quantification results of control cells and Huh7 STX6-OE cells treated with p-STAT3 inhibitor or DMSO in Transwell migration assays. *n* = 6 per group. (**D**) Protein levels and quantification results of PCNA, cyclin D1, E-cadherin and N-cadherin in control and Huh7 STX6-OE cells treated with p-STAT3 inhibitor or DMSO. *n* = 6 per group. β-actin served as the loading control. Data was given as mean ± SD. For statistical analysis, the two-tailed Student’s *t*-test was used in **A**-**D**. n.s. indicates no significant difference; * *P* < 0.05; ** *P* < 0.01

To explore the dependence of STX6 function on STAT3 activity, the inhibitor of STAT3 activity was applied to STX6-OE and control cells. WB analysis revealed that STX6 promoted the expression of p-STAT3 but not the total STAT3 in the cells, while the inhibitor markedly decreased the expression of p-STAT3 both in the control and STX6-OE expression cells **(**Fig. 6A**)**, which indicated the efficacy of the inhibitor and the dependence of STX6 function on STAT3 activity. Further, the colony formation and Transwell assays were performed to validate the above results. As shown, in Huh7 cells, the inhibitor of p-STAT3 blocked the promotive effects of STX6 overexpression on the cell proliferation **(**Fig. 6B**)**. Transwell migration assay indicated that the inhibitor reversed the STX6 overexpression-enhanced Huh7 cell migration **(**Fig. 6C**)**. Moreover, p-STAT3 inhibitors abolished the protein increases of PCNA, cyclin D1, and N-cadherin expression and the protein reduction of E-cadherin due to STX6 expression **(**Fig. 6D**)**. Overall, these results proved that STX6 exerts its function in HCC progression through increasing STAT3 phosphorylation.

## Discussion

Previously, *STX6* has been shown to be upregulated in several types of tumors, including HCC, colorectal cancer, pancreatic ductal adenocarcinoma, and esophageal cancer [14, 16–19]. Consistent with these studies, we found that *STX6* was highly expressed in HCC tissues compared with paracancerous tissues and the protein level of STX6 was also increased in cancerous tissues. Besides, one report had pointed out that HCC cells overexpressing USF2 (upstream stimulatory factor 2) had decreased expression level of STX6, and USF2 could bind to the promoter of STX6 by three binding motifs, proving that in HCC cells, USF2 functioned as transcriptional repressor of STX6 [18]. However, other regulatory mechanisms by which STX6 plays an oncogenic role in HCC need to be further investigated.

STX6 has been reported to contribute to the development of multiple tumors. In esophageal squamous cell carcinoma, STX6 knockdown inhibited cell cloning and migration by up-regulating p53 expression and regulating integrin transport [14]. STX6 was found to be highly expressed in prostate cancer and increased the production of enzalutamide-resistant cells in prostate cancer by regulating exosomal secretion [17]. In papillary renal cell carcinoma, STX6 promotes tumor tissue angiogenesis, cell proliferation, and migration [15, 26], possibly by regulating VEGFR2. STX6 is upregulated in HCC and colorectal cancer, and knockdown of STX6 suppresses cell proliferation, migration, invasion, and cell cycle progression of BEL-7404 HCC cells and HCT116 colorectal cancer cells [16]. In HCC, *STX6* expression was significantly correlated with tumor size, Edmondson grade, alpha-fetoprotein level and poor prognosis. Furthermore, it was found that STX6 may be involved in the inflammatory response of HCC by binding to CD163, an anti-inflammatory factor secreted by M2 macrophages [19]. Recent studies have implicated that STX6 overexpression promotes HCC cell proliferation and metastasis in vivo and in vitro, and accelerates the degradation of microtubule-associated protein 1 light chain 3β (LC3) by stimulating autophagosome and lysosome binding, ultimately contributing to HCC progression [18]. Consistent with the above studies, we found that in HCC cell lines (Huh7 and HepG2), upregulation of *STX6* promoted proliferation, migration and invasion, while STX6 knockdown showed the opposite results. In addition, the results of tumorigenic assays in nude mice indicated that STX6 was positively correlated to the growth and metastasis of HCC cells. These results suggest that STX6 plays a driving role in the development of HCC.

To further explore the molecular mechanism of STX6, the RNA-Seq analysis of STX6 knockdown HepG2 cells and its control cells showed that the JAK-STAT signaling pathway was the most significantly enriched pathway leading to STX6-mediated phenotypic changes. The STAT transcription factor family includes STAT1, STAT2, STAT3, STAT4, STAT5a, STAT5b, and STAT6 [27]. STAT proteins are involved in tumor cell proliferation, inhibition of apoptosis, promotion of tumor cell stemness, and chemoresistance by transmitting signals from cytokines, growth factors, and their receptors [28, 29]. Among them, STAT3 was perceived to be a critical promoter of cancer progression. Previous researches have shown that STAT3 is hyperactivated in various tumor behaviors, inducing tumor growth, angiogenesis, immunosuppression, and tumor invasion [30–32]. In multiple myeloma, activated STAT3 inhibits apoptosis by regulating the transcription of the Bcl-x gene promoter [33]. In lung cancer, the JAK2/STAT3 signaling pathway is regulated by SETD7 and IL-6 levels that affect lung cancer cell migration, invasion and brain metastasis [34, 35]. In colorectal cancer, STAT3 binds to NF-κB in response to IL-6 and TNF-α stimulation which promotes cancer cell invasion [36]. STAT3 and NF-κB promote breast cancer metastasis by inducing the expression of the key regulator Fascin [37]. Nitidine chloride is crucial in inhibiting angiogenesis and tumor growth in gastric cancer through inhibition of the STAT3 signaling pathway [28]. It has also been shown that STAT3 is involved in miR-340-5p regulating the migration process of HBV-HCC cells and exacerbates TGF-β1-induced EMT and migration of hepatocytes [38, 39]. In this study, overexpression of STX6 promoted STAT3 phosphorylation, and knockdown of STX6 resulted in the opposite effects. Moreover, p-STAT3 inhibitor significantly reduced the promotive effects of STX6 overexpression on HCC proliferation and metastasis. These results proved that STX6 promoted HCC development by activating the STAT3 signaling pathway.

## Conclusions

In summary, this study revealed that STX6 was highly expressed in HCC tissues. The experiments in vitro and in vivo proved that STX6 promoted HCC cell proliferation, migration and invasion by regulating the STAT3 signaling pathway. Mechanistically, inhibition of STAT3 phosphorylation abolished STX6-mediated promotive effects on HCC progression. Our study confirmed the oncogenic role of STX6 in hepatocellular carcinoma, suggesting that STX6 may be a potential target for hepatocellular carcinoma treatment.

### Electronic supplementary material

Below is the link to the electronic supplementary material.

Supplementary Material 1

## Data Availability

All data generated or analyzed during this study are included in this published article and its supplementary information files.

## Declarations

### Author information

Li Huang and Xiaoting Zhong contributed equally to the study.

### Authors and affiliations

Department of oncology, First Affiliated Hospital, Gannan Medical University; Jiangxi Clinical Medical Research Center for Cancer, Ganzhou, 341000, China.

Li Huang, Xiaoting Zhong, An Li, Fuping Tu, Miao He, Xueming Xu, Xiaohui Liu, Xiaoli Zeng, Jun Chi, Xiangcai Wang & Jianming Ye.

Gannan Innovation and Translational Medicine Research Institute, Gannan Medical University, Ganzhou, 341000, China.

Tian Tian.

Department of critical medicine, First Affiliated Hospital, Gannan Medical University, Ganzhou, 341000, China.

Chunli Wang.

### Additional file 1

Raw Data.

## Abbreviations

STX6: Syntaxin6
SNAREs: Soluble N-ethylmaleimide-sensitive factor attachment protein receptors
HCC: Hepatocellular carcinoma
RACK1: Receptor for activated protein kinase C
JAK: Janus tyrosine Kinase
STAT: Signal Transducer and Activator of Transcription
HBV: Hepatitis B virus
HCV: Hepatitis C virus
v-SNAREs: Vesicle Soluble N-ethylmaleimide-sensitive factor attachment protein receptors
t-SNAREs: Target Soluble N-ethylmaleimide-sensitive factor attachment protein receptors
TNF-α: Tumor necrosis factor-α
ShRNA: Short hairpin RNA
TCGA: The Cancer Genome Atlas
cDNA: Complementary DNA
PVDF: Polyvinylidene difluoride
DAB: Diaminobenzidine
CCK-8: Cell Counting Kit-8
PBS: Phosphate buffer solution
GSEA: Gene set enrichment analysis
DEGs: Differentially expressed genes
KEGG: Kyoto Encyclopedia of Genes and Genomes
SPSS: Statistical Product and Service Solutions
PCR: Polymerase Chain Reaction
OS: Overall survival
WB: Western Blot
IHC: Immunohistochemistry
STX6-OE: Overexpressing Syntaxin6
shSTX6: Syntaxin6 knockdown
PCNA: Proliferating cell nuclear antigen
cyclin D1: Cell cycle protein D1
E-CAD: E-cadherin
N-CAD: N-cadherin
EMT: Epithelial-mesenchymal transition
IP: Immunoprecipitation
IP-MS: IP coupled with mass spectrometry
p-STAT3: Phosphorylated signal transducer and activator of transcription 3
LC3: light chain 3
VEGFR2: Vascular Endothelial Growth Factor Receptor 2
IL-6: Interleukin-6
NF-κB: Nuclear factor kappa-B
USF2: upstream stimulatory factor 2
