# Supplementary material for: Syntaxin6 contributes to hepatocellular carcinoma tumorigenesis via enhancing STAT3 phosphorylation
Source: Cancer Cell Int. 2024 Jun 4;24:197. doi: 10.1186/s12935-024-03377-3 (PMC11149193; doi:10.1186/s12935-024-03377-3)

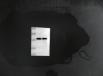

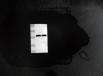

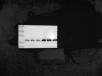

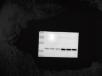

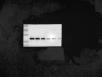

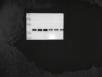

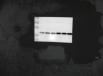

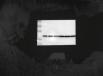

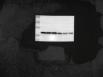

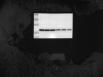

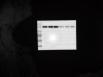

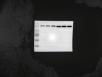

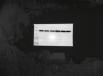

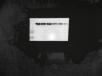

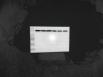

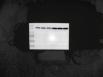

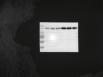

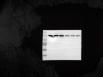

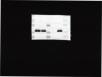

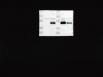

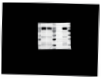

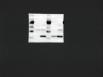

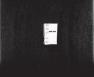

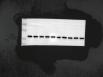

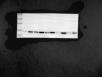

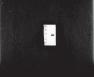

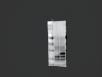

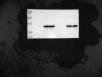

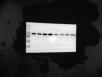

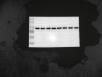

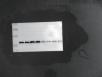

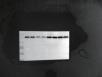

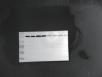

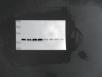
**Figure 2**

HepG2-OE

**Figure 1 F**

**A** Huh7-OE


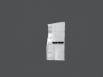


**Figure 2**

**D** Huh7 overexpression proliferative marker


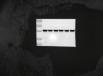


HepG2-SH

**E** Huh7-SH


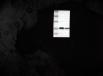


**H** Huh7 knockdown proliferation marker assay


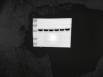


HepG2 overexpression proliferative marker


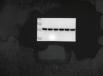


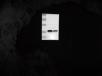


HepG2 knockdown proliferation marker assay


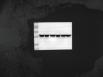


**Figure 3**

**D**HepG2 overexpression migration marker detection


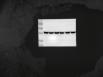


**I** HepG2-shSTX6-migration marker detection


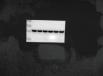


**E**Huh7 overexpression migration marker detection


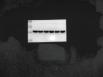


**J** Huh7-shSTX6-migration marker detection


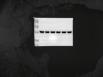


**Figure 4**

**B**


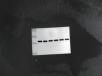


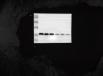


**G**


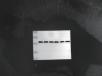


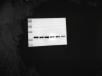


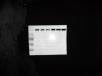


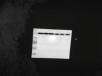


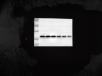


**Figure 5**

**G**

IP-HA

(right）IN-H-HA-AM

IP-FLAG

(left)IN-F-FLAG-AM

**H**

IP-HA

(right）IN-H-HA-AM

IP-FLAG

(left)IN-F-FLAG-AM

**I**


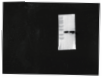


IN-H-FLAG-AM


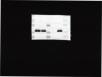


(left)IN-F-HA-AM


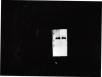


IN-H-FLAG-AM


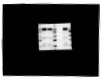


(left)IN-F-HA-AM

**J**


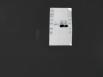


IP-H-FLAG-AM


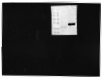


IP-F-FLAG-AM


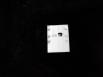


IP-H-FLAG-AM


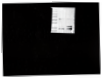


IP-F-FLAG-AM


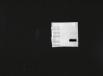


IP-H-HA-AM


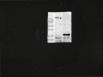


IP-F-HA-AM


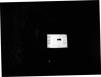


IP-H-HA-AM


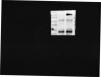


IP-F-HA-AM


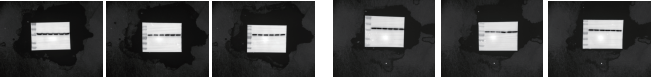


**K**


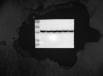


**M**


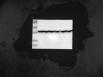


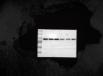


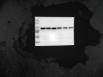


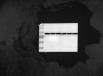


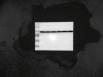


**L**


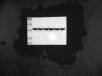


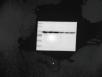


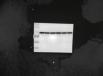


**Figure 6**

**A**


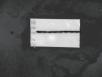


**D**


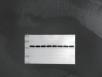

Supplement: Supplementary file 1 — Supplementary Material 1 [file 12935_2024_3377_MOESM1_ESM.docx]
